# Pheochromocytoma metastasis to the central nervous system: a case report and systematic review

**DOI:** 10.3389/fendo.2025.1633411

**Published:** 2025-08-25

**Authors:** Justyna Małgorzata Fercho, Oskar G. Chasles, Jakub Chamier-Gliszczyński, Dominik Ryszard Płaza, Bogdan Jabłoński, Klaudia Kokot, Maciej Mielczarek, Małgorzata Małłek-Grabowska, Jacek Szypenbejl, Adrian Szkudlarek, Tomasz Szmuda, Mariusz Siemiński, Jacek Furtak

**Affiliations:** ^1^ Neurosurgery Department, 10th Military Research Hospital and PolyClinic SPZOZ, Bydgoszcz, Poland; ^2^ Department of Emergency Medicine, Medical University of Gdańsk, Gdańsk, Poland; ^3^ Lung Transplant Unit, Cardiac Surgery Department, Medical University of Gdańsk, Gdańsk, Poland; ^4^ Scientific Circle of Neurotraumatology, Department of Emergency Medicine, Medical University of Gdańsk, Gdańsk, Poland; ^5^ Neurosurgery Department, Stanisław Staszic Specialist Hospital, Piła, Poland; ^6^ Department of Anesthesiology and Intensive Therapy, 10th Military Research Hospital and Polyclinic SPZOZ, Bydgoszcz, Poland; ^7^ Faculty of Medicine, Bydgoszcz University of Science and Technology, Bydgoszcz, Poland; ^8^ Faculty of Medicine, Medical University of Gdańsk, Gdańsk, Poland

**Keywords:** pheochromocytoma, malignant pheochromocytoma, spinal metastases, cerebral metastases, central nervous system

## Abstract

**Background:**

Pheochromocytoma (PCC) is a rare neuroendocrine tumor, with 10–15% of cases showing malignant behavior defined by metastatic spread, including exceptionally rare central nervous system (CNS) involvement. Brain metastases present unique diagnostic and therapeutic challenges due to their potential to impair neurological function. This study reports a case of malignant PCC (mPCC) with CNS metastases and a systematic review to clarify the clinical patterns, management strategies, and prognostic factors.

**Methods:**

We describe the surgically managed case of a 41-year-old man with right frontoparietal brain metastasis. A systematic review, adhering to the PRISMA 2020 guidelines, searched PubMed, Scopus, and Web of Science for peer-reviewed studies on mPCC with brain or spinal metastases confirmed by radiology or histopathology. Data on demographics, symptoms, imaging, treatments, and outcomes were extracted and descriptively analyzed using Python-generated graphics.

**Results:**

This review identified 18 cranial (1948–2022) and 60 spinal (1977–2024) metastasis cases from 53 studies. Cranial metastases were present at a mean age of 46.6 years (SD 14.1), commonly with headaches (44.4%) and neurological deficits, such as weakness, presented in our case, with 72.2% surgically treated. Spinal metastases occurred at a mean age of 44.5 years (SD, 16.0), often with hypertension (51.7%) or pain, with a mean of 1.7 lesions (SD 1.5). The patient achieved short-term symptom relief post-resection, but incomplete follow-up (33.3% cranial) and reporting gaps (63.3% spinal laterality) limited the prognostic insights. MRI and PET improved the diagnostic accuracy over historical non-contrast CT use (41.7% spinal cases).

**Interpretation:**

CNS mPCC metastases are exceedingly rare with distinct neurological (cranial) and structural (spinal) presentations. Advanced imaging, particularly magnetic resonance imaging (MRI) and positron emission tomography (PET), is critical for accurate diagnosis and surgical planning. Sparse data underscores the need for registries and prospective studies to standardize care and improve outcomes.

## Introduction

Pheochromocytoma (PCC) is a rare neuroendocrine tumor arising from chromaffin cells, predominantly in the adrenal medulla, with an estimated incidence of 0.8 per 100,000 person-years (1). Alongside extra-adrenal paragangliomas, PCC forms a spectrum of catecholamine-secreting tumors that affect patients globally, with variations in prevalence across sporadic and hereditary contexts. Its pathophysiology centers on excessive catecholamine production, including epinephrine, norepinephrine, and dopamine, manifesting as paroxysmal hypertension, tachycardia, diaphoresis, and, in extreme cases, hypertensive crises that threaten cardiovascular stability (1). PCC occurs sporadically or within hereditary syndromes like von Hippel-Lindau disease (VHL), multiple endocrine neoplasia type 2 (MEN2), or neurofibromatosis type 1 (NF1), with mutations, particularly SDHB, increasing malignancy risk (2, 3).

Approximately 10–15% of PCCs are malignant, defined by metastatic spread, with a slight male predominance noted in some series (4). Common metastatic sites include the liver, lungs, and skeleton; however, central nervous system (CNS) involvement, encompassing brain and spinal metastases, is extraordinarily rare, with fewer than 100 cases reported over eight decades (5, 6). The scarcity of CNS metastases complicates diagnosis and management, as their neurological consequences (e.g., deficits and seizures) and structural impacts (e.g., spinal instability) require specialized intervention.

Diagnosis of malignant PCC (mPCC) integrates biochemical assays, imaging, and genetic profiling. Plasma-free metanephrines and urinary catecholamines offer high sensitivity, although false positives require careful interpretation (7, 8). Imaging modalities such as computed tomography (CT), magnetic resonance imaging (MRI), and 123I-metaiodobenzylguanidine (MIBG) scintigraphy localize tumors, with MRI excelling for CNS lesions and positron emission tomography (PET) aiding systemic staging (9). Genetic testing identifies high-risk mutations; however, global disparities limit access (2). Management hinges on preoperative alpha- and beta-adrenergic blockade to stabilize hemodynamics, followed by surgery for resectable metastases. CNS cases require additional consideration, balancing neurological preservation and oncological control (5, 10).

This study presents a rare case of CNS mPCC cerebral metastasis, illustrating distinct clinical and therapeutic complexities. The patient signed an informed consent form, allowing the authors to publish the results. Through a systematic review, we synthesized global data to elucidate the demographics, diagnostics, treatments, and gaps, aiming to inform clinical practice and future research on this understudied malignancy.

## Case report

### Cerebral metastasis

#### Patient background

A 41-year-old right-handed male was referred to the Neurosurgery Department for a right frontoparietal tumor identified by computed tomography (CT) at Głogów Hospital after two weeks of progressive left-sided weakness. His history included mPCC diagnosed post-laparoscopic adrenalectomy (September 2007) with open resection of a local recurrence (July 2008). Exploratory laparotomy (June 2015) revealed unresectable inferior vena cava invasion. He received stereotactic radiotherapy to the adrenal bed (30 Gy, May 2017), followed by 177Lu-DOTATATE for retroperitoneal lymph node metastases (first dose, November 2022), and sunitinib (initiated on March 9, 2023), achieving a partial response. The comorbidities included resistant hypertension, likely catecholamine-driven, and suspected autoimmune thyroiditis. To date, no allergies have been reported. The patient was later referred to the Neurosurgery Department at the 10^th^ Military Research Hospital and Polyclinic in Bydgoszcz, Poland, due to the need for further treatment requiring greater neurosurgical capabilities. Enabling this treatment necessitated the patient’s transport across two voivodeships.

#### Clinical findings

On admission, the patient was alert and oriented, with a Glasgow Coma Scale (GCS) score of 15 and Karnofsky Performance Status (KPS) score of 80, reflecting preserved function despite systemic disease. Neurological examination revealed no motor deficits, although prior weakness prompted imaging.

#### Preoperative diagnosis

Suspected mPCC metastasis to the right frontoparietal region based on CT and oncological history.

#### Surgical management

On August 1, 2024, neuronavigation-assisted craniectomy was performed under general anesthesia. The patient was supine, and the head was secured using the Z-touch neuronavigation system to map the tumor. A parietal incision enabled a skin-fascia flap and craniectomy near the sagittal sinus, which was controlled with hemostatic sutures. Dural incision revealed no initial motor cortex response upon stimulation. A 5-mm cortical incision exposed a grayish-cream, moderately vascular tumor resected via a cavitron ultrasonic surgical aspirator (CUSA). A second brownish, highly vascular segment extended into the right lateral ventricle, requiring meticulous resection. Fluorescent residual tissue (pink under UV light) was cleared. Stimulation (5 mA) at the anteromedial margin elicited left extremity responses, indicating motor cortex proximity, prompting careful closure. Hemostasis was performed using Surgicel; the dura was sealed with sutures and TachoSil, and the bone flap was secured with CranioFix, followed by cranioplasty with bone cement. The wound was closed in layers and the patient was transferred to the ICU. Preoperative alpha- and beta-blockers ensured hemodynamic stability with no intraoperative complications.

#### Histopathology

Two samples confirmed metastatic mPCC, showing a nested (zellballen) architecture typical of pheochromocytoma. Immunohistochemistry revealed the following findings:

- Positive: Vimentin (++, strong), CD10 (+, weak), Chromogranin A (++, strong), and synaptophysin (++, strong).
- Negative: GATA3, Melan A, CAM 5.2, TTF1, CK-PAN, and S-100.

Strong positivity (++) for Chromogranin A and Synaptophysin, with weaker CD10, confirmed a neuroendocrine origin, ruling out carcinoma or melanoma. The absence of GATA3 and TTF1 excluded thyroid or lung primaries, aligning with the patient’s history of mPCC.

#### Postoperative imaging

**Figure 1:**
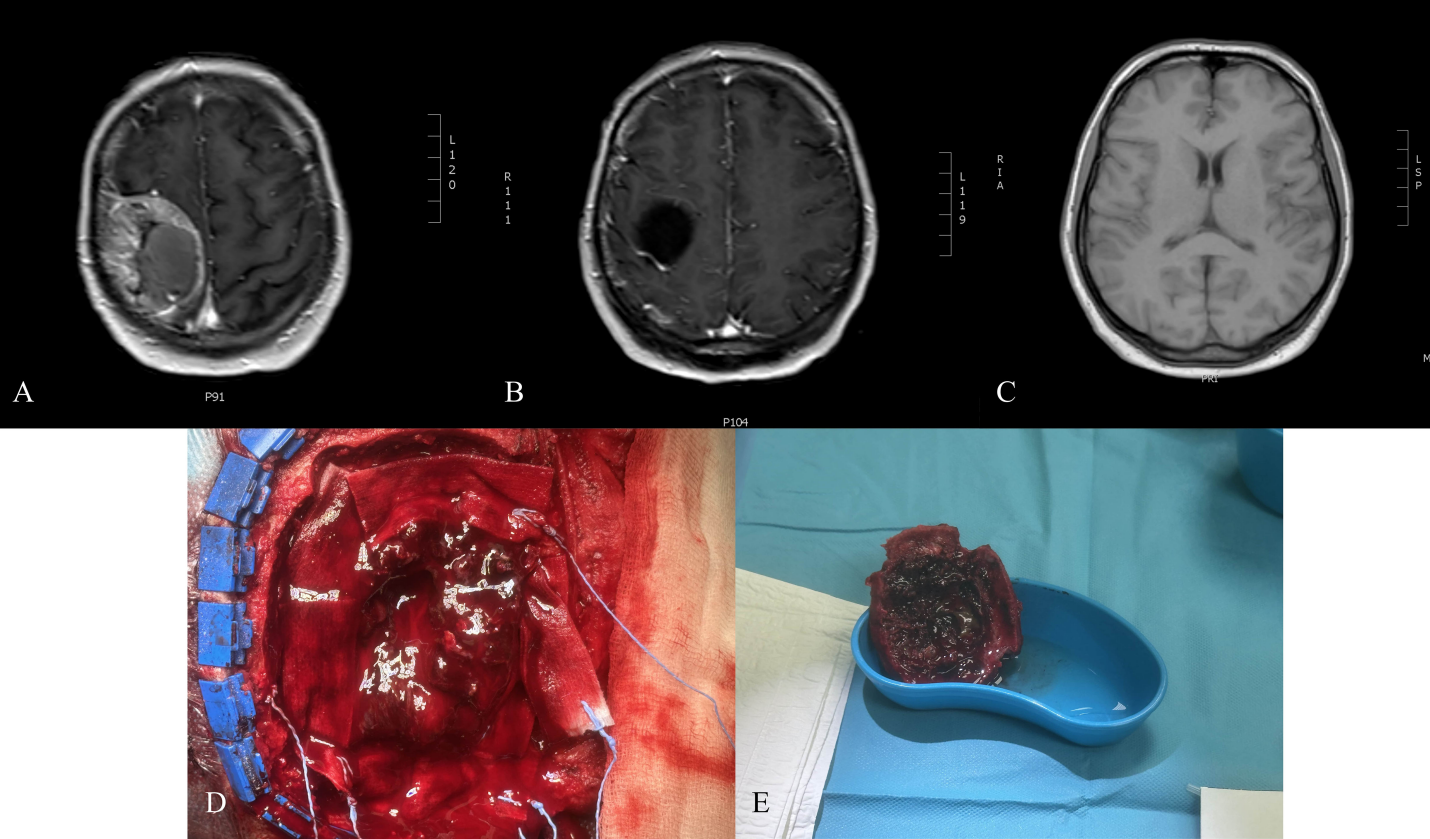
**(A)** Preoperative T1-weighted MRI with contrast showing cranial metastasis of the pheochromocytoma. **(B)** Non-contrast CT scan performed on the first postoperative day, demonstrating postsurgical changes. **(C)** T1-weighted MRI with contrast at 3 months postoperatively, illustrating the postoperative outcomes. **(D)** Intraoperative photograph of resected pheochromocytoma metastasis. **(E)** Intraoperative photograph obtained during the resection of a cranial metastatic tumor.

Non-contrast CT (August 2, 2024) showed a 48 × 23 mm fluid-filled resection cavity, consistent with postoperative changes, with successful cranioplasty. Brain parenchyma, ventricles, and midline were unremarkable (**Figure 1**).

#### Postoperative diagnosis

Resected right frontoparietal mPCC metastasis with cranioplasty, histologically verified.

#### Outcome

Short-term neurological preservation was achieved with no immediate deficits, although systemic disease indicated a guarded prognosis.

## Methodology

### Search strategy and protocol

**Figure 2:**
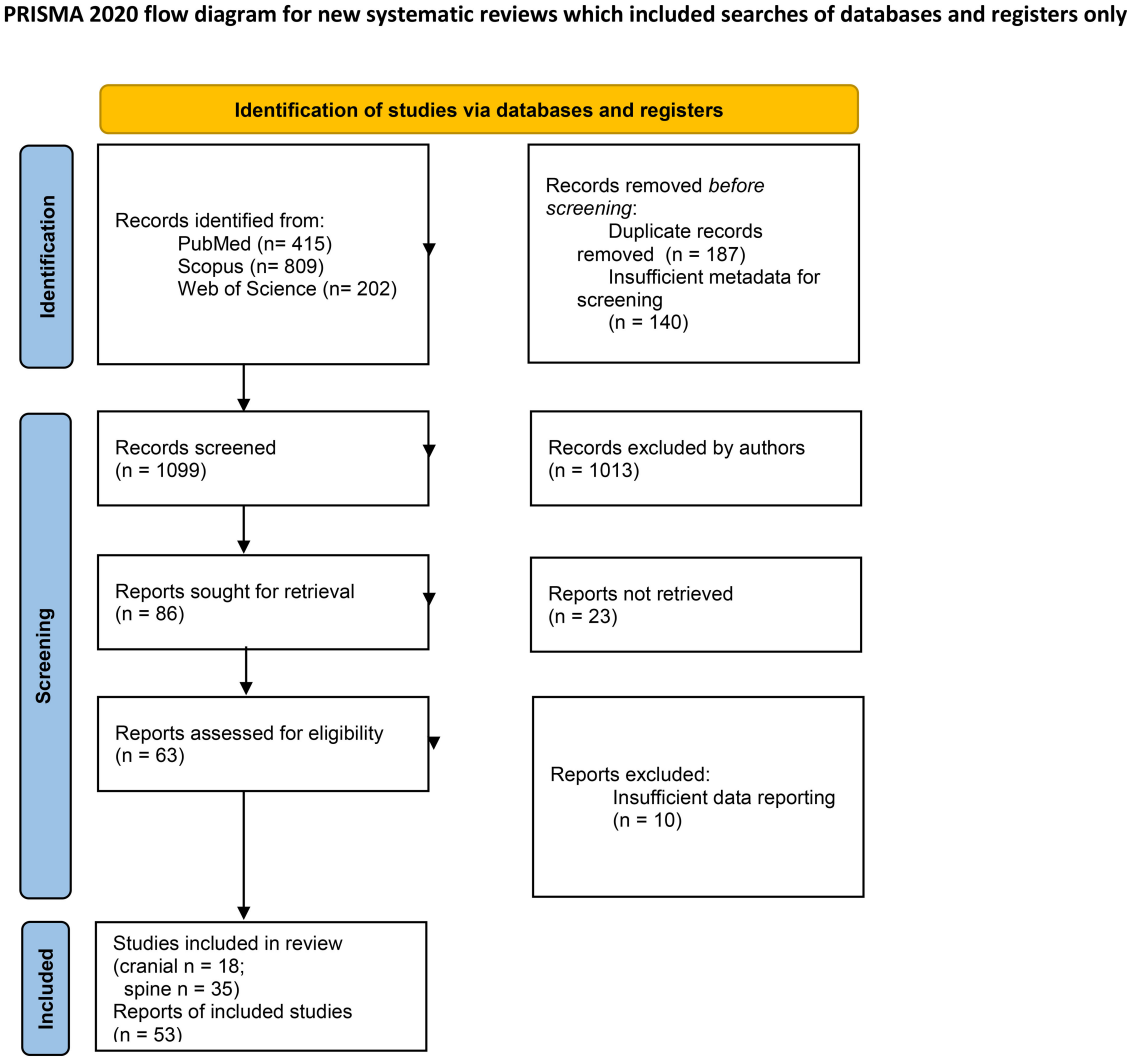
PRISMA Flowchart.

This systematic review was conducted in accordance with PRISMA 2020 guidelines. A comprehensive search strategy was developed and implemented across three electronic databases, PubMed, Scopus, and Web of Science. The search utilized the following terms: (“pheochromocytoma”) AND ((“malignant”) OR (“metastasis”)) AND ((“brain” OR “cerebrum” OR “intracranial” OR “cranium” OR “skull”) OR (“spine” OR “vertebra”)). No date or language restrictions were applied, resulting in 1426 records from 1948 to 2024. After removing 187 duplicates and 140 results owing to insufficient metadata for screening, 1099 unique records were retained for screening (**Figure 2**).

### Selection criteria

The eligibility criteria were peer-reviewed case reports and case series documenting patients with primary adrenal pheochromocytoma (PCC) and metastatic lesions to the central nervous system or related bony structures (cranium or spine), who granted consent for publication to the authors of included studies. Metastatic disease requires confirmation via radiological (e.g., CT, MRI) or histopathological evidence. The exclusion criteria included studies with insufficient data (n = 10), studies unrelated to the specified anatomical regions, and studies with irretrievable full texts (n = 23). A two-stage screening process was conducted by four authors (OC, JG, DP, and BJ): initial title and abstract screening, followed by full-text analysis. Discrepancies during screening were resolved through consensus discussion or adjudication by senior authors (JF, MM, and TS). Manual screening was used exclusively, without artificial intelligence or automation tools, to ensure rigorous human oversight during the study selection.

### Statistical analysis

Fifty-three peer-reviewed studies, comprising 78 individual cases of metastatic adrenal PCC (18 cranial, 60 spinal), were included in this retrospective analysis. Extracted clinical data included patient factors (age at diagnosis, sex, genetic predisposition, von Hippel-Lindau disease, tumor laterality, number and location of metastases, admission symptoms, urinary metanephrine/normetanephrine levels, and immunohistochemical markers) and treatment factors (treatment method, follow-up duration, and outcomes). Descriptive statistics were calculated as follows: continuous data (e.g., age and number of metastases) were reported as means, standard deviations (SD), medians, and ranges; categorical data (e.g., genetic causes, imaging modalities) were expressed as proportions. Missing data (“not specified” [NS]) were noted as percentages where applicable. Data were tabulated and synthesized descriptively, providing extractable insights into the clinical course of the patients. No artificial intelligence methods were employed in the data extraction or analysis; all graphical presentations, including histograms, bar charts, and pie charts, were generated using Python with the matplotlib and seaborn libraries to ensure a clear and accurate representation of the findings.

## Results

### Cranial metastases

**Figure 3:**
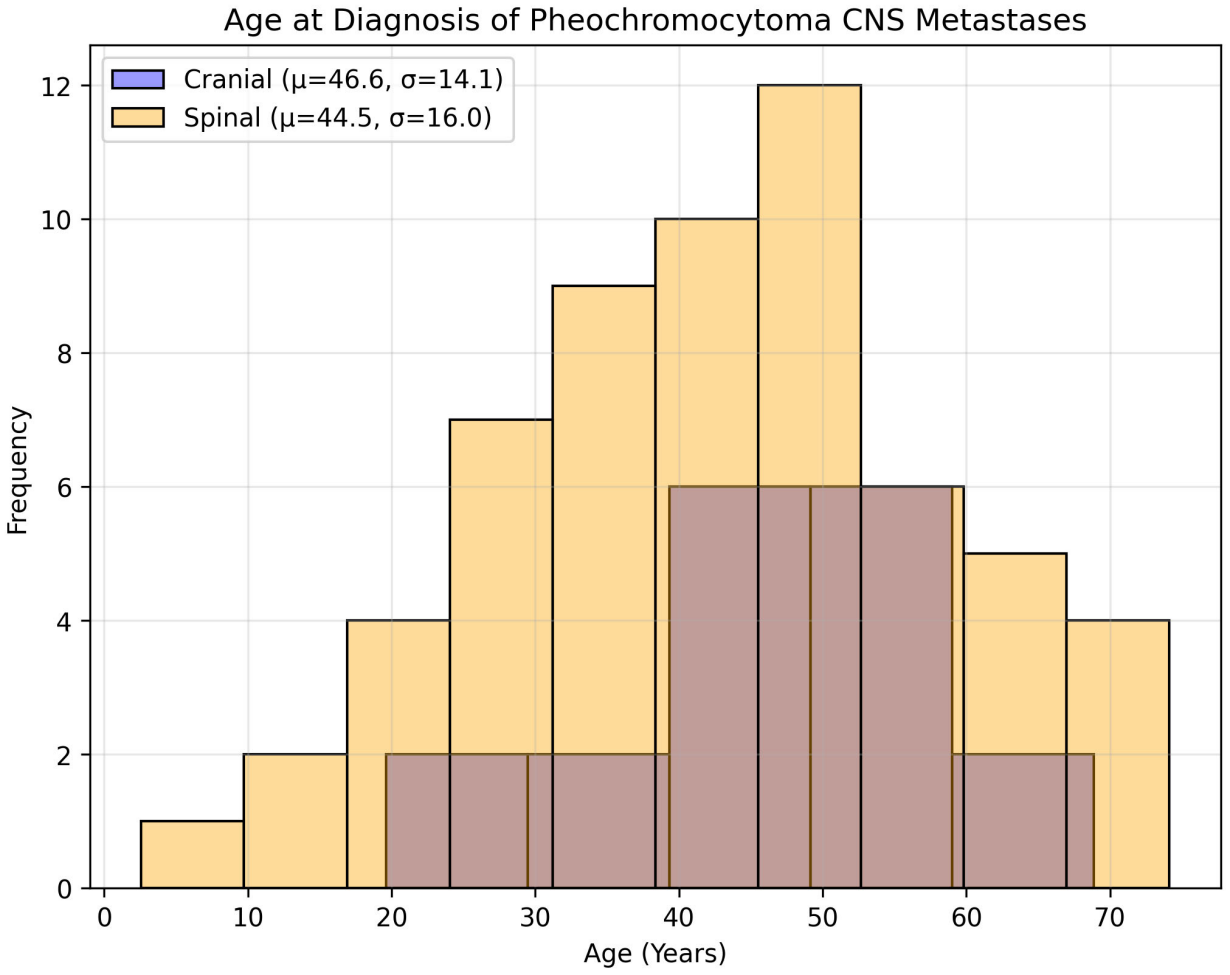
Age at diagnosis of pheochromocytoma CNS metastasis.

**Figure 4:**
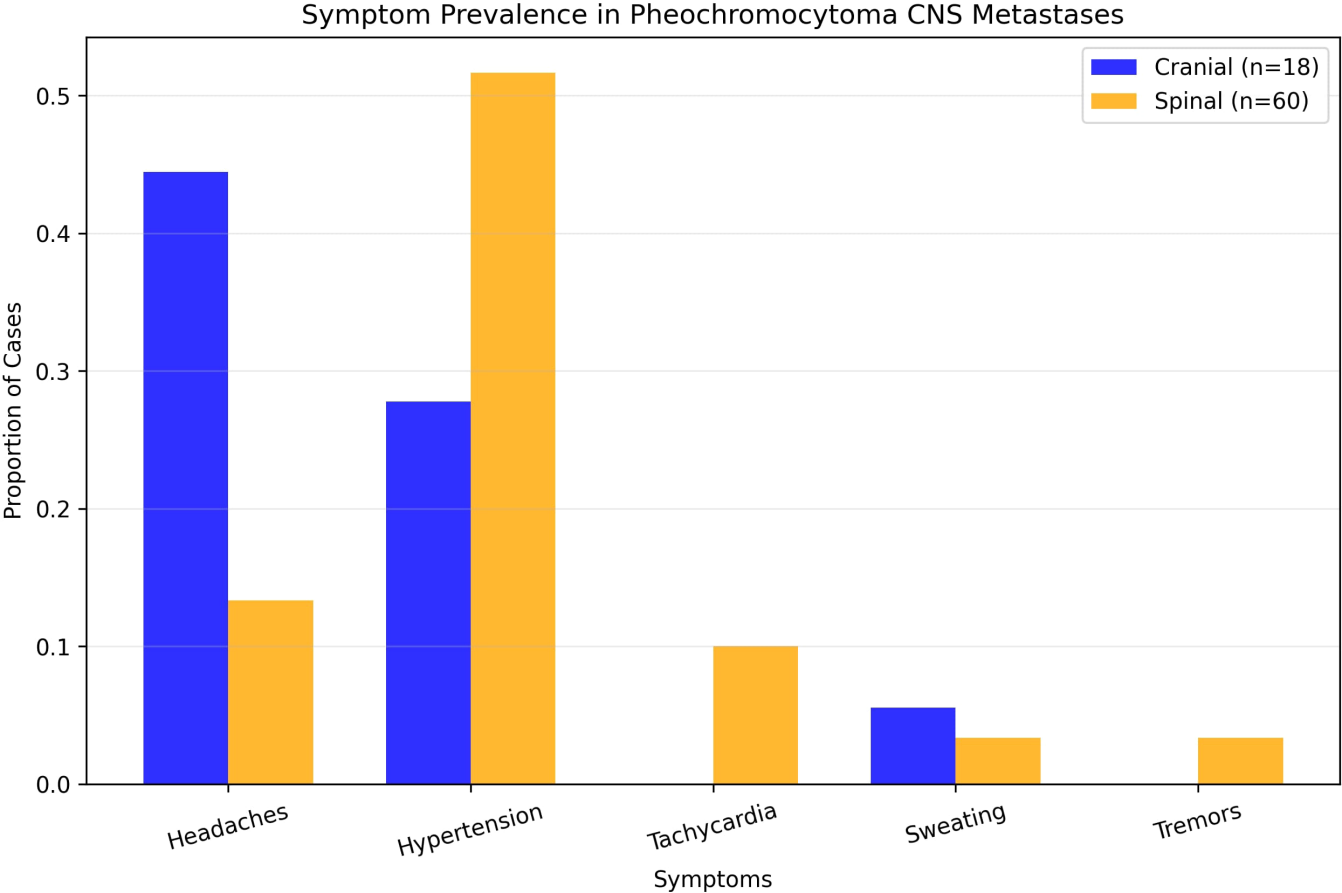
Prevalence of symptoms in pheochromocytoma CNS metastases.

Eighteen studies (1948–2022) reported 18 cases (10 males [55.6%] and 8 females [44.4%]) (**Table 1**). The mean age at PCC diagnosis was 35.0 years (SD 14.4, median 34, range 10–53); metastases occurred at 46.6 years (SD 14.1, median 48, range 23–73) (**Figure 3**), with an 11.6-year latency. Genetic predisposition was 16.7% (n=3, no VHL). The tumors were unilateral (72.2%, n=13), bilateral (5.6%, n=1), or unspecified (22.2%, n=4) (**Figure 4**). The metastases were solitary (n=10), dual (n=1), numerous (n=5), or unspecified (n=2). Symptoms included headache (44.4%, n=8), hypertension (27.8%, n=5), and sweating (5.6%, n=1). Treatments included resection (72.2%, n=13), radiotherapy (38.9%, n=7, including one MIBG, one stereotactic), or chemotherapy (11.1%, n=2); one case lacked data. Follow-up (12/18 cases, 33.3% missing) revealed 7 deaths, 1 relapse, and 4 no relapses. Immunohistochemistry revealed Chromogranin A (n=10), synaptophysin (n=7), and neuron-specific enolase (n=5).

**Table 1:** summarizes the data from 18 studies (1948–2022, 18 patients) on cranial pheochromocytoma metastases, including demographics, symptoms, imaging, treatment, immunohistochemistry, and outcomes.

Reference	Sex	Age at initial diagnosis	Laterality	Age at resection/adrenalectomy	Age at metastasis diagnosis	Location of cranial metastasis	Symptoms	Treatment	Immunohistochemistry results
(11)	F	NS	Bilateral	No adrenalectomy	51	Peduncles, cerebellum, pons, cerebrum	Headaches, syncope, poor memory, drowsiness, convulsions	Right subtemporal decompression	NS
(12)	M	NS	Unilateral	NS	61	NS	Hypertension, sweating, headaches, convulsions, palpitations	None	NS
(13)	M	NS	NS	NS	47	Cerebrum, cerebellum	Cognitive deterioration	None	NS
(14)	F	43	Unilateral	NS	43	Prominent extradural scalp mass in parietal region	Painless scalp mass, low back pain	Resection	chromogranin, neuron-specific enolase
(15)	F	NS	Unilateral	41	49	NS	Hypertension	NS	NS
(16)	F	NS	NS	NS	31	Right frontal lesion	Partial motor seizure	Resection	Neuron-specific enolase, S100 protein and chromogranin, negative anti-calcitonin
(17)	M	37	Unilateral	37	47	Right frontotemporal	Hypertension	Resection, MIBG therapy	Chromogranin, negative progesterone receptors and epithelial membrane antigen
(18)	F	NS	NS	23	46	Right temporoparietal extra- axial lesion with inner table skull infiltration	Hypertension, headaches, vomiting	Resection	Neuron-specific enolase, chromogranin A, synaptophysin
(19)	F	NS	Unilateral	59	60	Left frontal and parietal lobe	Hypertension	Resection, radiotherapy	NS
(20)	M	49	Unilateral	49	54	Left occipital lobe	Headaches, cognitive decline, visual loss, shortness of breath	Resection, pre-op chemotheray, post-op radiotherapy	Neuron specific enlase, chromogranin A, negative keratin, somatostatin, S-100
(21)	F	19	NS	NS	23	Left occipital, parietal, right frontal lobes	Altered mental status	None	NS
(22)	M	53	Unilateral	53	73	Right parietal lobe	Loss of cognitive function, ahomonymous hemianopsia and left sided dysmetria	Resection, whole brain radiotherapy	NS
(23)	M	29	Unilateral	29	57	Left parietal lobe	Headachesm right hemiparesis, aphasia, dysartria	Pre-op stereotactic radiosurgery,resection	Chromagranin A, synaptophysin, S100
(24)	M	10	Unilateral	11	24	Right posterior epidural parietal region	None	Resection	Synaptophysin, chromogranin
(25)	M	52	Unilateral	51	52	Left parietal lobe, bilateral cerebral hemisphere, right pons	Headaches dizziness, motor aphasia	Resection of largest lesion, post-op whole brain radiotherapy	Synaptophysin, S-100, negative Oligo-2
(26)	F	27	Unilateral	27	29	Extradural lesion attached to the tentorium	Headaches, vomiting, static and kinetic cerebellar syndrome	Resection, post-op whole-body radiotherapy	Synaptophysin, chromogranin, neuron-specific enolase
(27)	M	NS	Unilateral	55	60	Left temporal lobe	Scalp mass	Resection	Vimentin, Syn and CgA, negative S-100, EMA and CD10
(28)	M	31	Unilateral	31	31	Right frontal bone	Elevated urine metanephrine on routine check-up	Resection	chromogranin, synaptophysin
Presented case	M	24	Unilateral	24	41	Right frontotemporal region	Hypertension, progressive left-sided weakness	Resection	Vimentin, CD10, chromogranin A, synaptophysin, negative GATA3, Melan A, CAM 5.2, TTF1, CK-PAN, S-100

NS, not specified.

### Spinal metastases

Thirty-five studies (1944–2024) reported 60 cases (**Table 2**). The mean age at PCC diagnosis was 40.5 years (SD, 15.4; median, 42; range, 11–68 years), and metastases occurred at 44.5 years (SD 16.0, median 44, range 9–74) (**Figure 3**), with a 4-year latency. Adrenalectomy occurred in 36.7% (n=22, mean age 42.1 years, SD 18.3), 13.3% (n=8) had none, and 50% (n=30) were unspecified. Genetics included an 8.3% predisposition (n=5, VHL 1.7% [n=1], NF1 3.3% [n=2]). The laterality was unspecified (63.3%, n=38), unilateral (31.7%, n=19), or bilateral (5%, n=3). The metastases were multiple (53.3%, n=32) or mean 1.7 lesions (SD 1.5, median 1, range 1–7). A total of 72 spinal metastases were recorded, nearly half of which were located in the thoracic spine (47.2%, n=34). No strong linear correlation was found between the co-occurrence of multiple tumors (**Supplementary Correlation Matrix 1**). The symptoms included hypertension (51.7%, n=31), headaches (13.3%, n=8), tachycardia (10%, n=6), and sweating/tremors (3.3%, n=2 each) (**Figure 4**). Imaging included non-contrast CT (41.7%, n=25), contrast CT (6.7%, n=4), non-contrast MRI (31.7%, n=19), contrast MRI (16.7%, n=10), and PET (21.7%, n=13), for a total of 71 modalities.

**Table 2:** details the data from 35 studies (1977–2024, 60 patients) on spinal pheochromocytoma metastases, including patient characteristics, symptoms, imaging findings, metastasis details, treatments, and outcomes.

Reference	Sex	Age at initial diagnosis	Laterality	Age at resection/adrenalectomy	Age at metastasis diagnosis	Location of spinal metastasis	Symptoms	Treatment	Immunohistochemistry results
(29)	M	14	unilateral	no adrenalectomy	14	T5, T6 vertebral body	Hypertension, partial paraplegia	Laminectomy, subtotal resetion, chemotherapy	NS
(30)	F	NS	NS	No adrenelectomy	39	C2-C3	Hypertension, headaches, hemiplegia, palpitation, dizziness, nausea, mild cardiomegaly, Babinski’s sign, retinal bleeding, papillary edema	Symptomatic treatment	NS
(31)	M	64	Unilateral	64	65	T7 body and pedicles	Hypertension, anorexia, constipation, weight loss	Laminectomy	NS
(32)	M	NS	NS	NS	38	C6 body	NS	Percutaneous embolization of the tumor	NS
(33)	F	47	Unilateral	47	51	S1-S4	Hypertension	Radiotherapy	NS
(34)	M	15	Bilateral	15	27	T2	Hypertension, paraparesis with spasticity, positive Babinski's sign bilaterally, blindness of the right eye and diminished vision of the left eye	Surgery	Synaptophysin
(35)	M	53	NS	53	65	T11	Left abdominal pain	Radiotherapy	Chromogranin A
(36)	M	47	Bilateral	47	47	T8	Hypertension, headaches, tremors, palpitation, flushing, shivers, urinary incontinence, anxiety attacks, pain and numbness, paraparesis hyperactive patella and achille reflexes, Babinski’s sign and clonus on the right side, hypoesthesy under T8 level	Laminectomy, resection	NS
(37)	M	68	Unilateral	68	69	C6-C7 body	Weakness, paresthesia of upper limbs	Vertebrectomy, stabilisation and plate fixation, resection, radiotherapy	NS
(38)	M	42	Unilateral	42	47	T2 body	Hypertension, urinary hesitancy, dysuria, weakness and numbness over the trunk and lower limbs, spastic paresis	Laminectomy, decompression, stereotactic radiotherapy	Synaptophysin, chromogranin
(39)	M	NS	unilateral	34	38	Lumbar spine	Pain	Percutaneous osteoplasty	NS
(40)	F	12	NS	NS	34	T9 body, pedicles, lamina	Hypertension, circumferential back pain	Fixiation, resection	NS
(41)	F	48	NA	No adrenalectomy	53	T1, T5, T10, T12 body	Hypertension, headache, tremors, palpitations, paresthesia, ftigue, flushing	Resection, fixation, cement-augmentation, auxiliary radiotherapy	NS
(42)	M	11	NS	NS	9	T10 body, pedicles	Hypertension, back pain	Laminectomy, sympathectomy, arthrodesis	NS
(43)	F	66	Unilateral	66	74	T10	Hypertension, pain in right hip with decreased range of motion	Radiotherapy, denied further intervention	NS
(44)	M	35	NS	NS	40	Entire axial spine	Axial spine pain, constipation	Cisplatin (200mg in total) injection	NS
(5)	M	28	NS	NS	NS	L3, L4	Abdominal and low-back pain	Embolization of the vascular supply	NS
(5)	M	41	NS	41	41	T1, T4–7, lumbar spine	Upper-back pain	Preoperative embolization, posterior C4–T10 instrumentation, anterior vertebral body resection	NS
(5)	F	21	NS	NS	21	Entire axial spine	Hypertension at the end of pregnancy	Posterior decompression and fusion, resection	NS
(5)	F	62	NS	68	66	Cervical spine, L1 body	Hypertension, palpitations, shortness of breath	Preoperative embolization of the spinal tumor, left lateral retroperitoneal corpectomy with resection, fusion	NS
(5)	M	23	NS	11	23	T-10 body	Hypertension, bloody stool and back pain	Embolization of intersegmental arteries, posterior fusion with vertebrectomy and cage placement	NS
(45)	F	56	Unilateral	NS	56	T7, L1, L5 pedicles, S2	Hypertension, headaches, lower back pain, right-sided sciatica, right foot drop	Decompressive laminectomy with tumor debulking, rhizolysis, fixation, radiotherapy	Synaptophysin, chromogranin A, CD17, CK5/6, plasma metanephrine and Ki-67
(46)	F	26	unilateral	no adrenalectomy	26	T8 body, T11-T12 body	Numbness and decreased muscle strength of lower limbs, urinary incontinence, lower back and hip pain	Posterior decompression, fixation, pedicle srews	Hematoxylin, eosin, chromogranin A staining, synaptophysin staining
(46)	F	26	unilateral (right)	NS	NS	T8, T11-T12	Headaches, acute numbness and decreased muscle strength of lower limbs, urinary incontinence	Palliative radiotherapy and chemotherapy, circumferential spinal cord decompression, stabilization	CgA, S-100,chromogranin, p53 and Syn;
(47)	M	54	unilateral	54	58	Sacrum body and posterior elements	Hypertension, tachycardia, progressive radiating back pain, right lower limb numbness	Embolization, cement augmentation, circumferential decopmression	Chromogranin A staining, synaptophysin staining
(47)	M	58	NS	NS	NS	Sacrum	Hypertension, progressive back pain, radiating pain, and numbness of right lower limb	Embolisation, osteoplasty, exploratory surgery, circumferential spinal cord decompression, stabilization, revision surgery	CgA, S-100,CD56, p53 and Syn;
(48)	F	48	Unilateral	48	66	T10 body	Hypertension, headaches, lower back pain, chest pain	Radiotherapy, chemotherapy	NS
(49)	F	31	NS	NS	NS	L3	Hypertension, paroxysmal headaches, low back pain	Dorsal instrumentation	CgA, S-100,CD56, NSE, vimentin and Syn;
(49)	M	58	NS	NS	NS	Sacrum	Hypertension, progressive low back pain, radiating pain and numbness of lower limbs	Dorsal instrumentation, cement augmentation, circumferential decompression	CgA, S-100,CD56, NSE, vimentin and Syn;
(49)	F	26	NS	NS	NS	T8, T11, T12	Paroxysmal hypertension, acute incomplete paralysis	Dorsal instrumentation	CgA, S-100,CD56, NSE, vimentin and Syn;
(49)	M	32	NS	NS	NS	T4	Progressive paraplegia and numbness of the lower limbs	Posterior decompression, resection, internal fixation	CgA, S-100,CD56, NSE, vimentin and Syn;
(49)	M	59	NS	NS	NS	T9, T10	Progressive back pain, numbness and decreased muscle strength of lower limbs	Posterior decompression, resection, internal fixation	CgA, S-100,CD56, NSE, vimentin and Syn;
(49)	M	63	NS	NS	NS	T2, T4, T7, L1, L3, sacrum	Hypertension, progressive lower back pain	Percutaneous vertebroplasty	CgA, S-100,CD56, NSE, vimentin and Syn;
(49)	M	27	NS	NS	NS	T1, T12, L1, L4	Back pain, decreased muscle strength of lower limbs	Percutaneous vertebroplasty, posterior decompression, resection internal fixation	CgA, S-100,CD56, NSE, vimentin and Syn;
(49)	F	22	NS	NS	NS	T5	Headache, back pain	Percutaneous vertebroplasty	CgA, S-100,CD56, NSE, vimentin and Syn;
(49)	M	60	NS	NS	NS	T11, L1 L3, L5	Numbness of both legs	Percutaneous vertebroplasty, posterior decompression, resection, internal fixation	CgA, S-100,CD56, NSE, vimentin and Syn;
(49)	M	38	NS	NS	NS	Sacrum	Sacrococcygeal pain	Biopsy, refused surgery	CgA, S-100,CD56, NSE, vimentin and Syn;
(50)	M	19	Unilateral	No adrenelectomy	19	Thoracic, lumbar, sarcal	Hypertension, tachycardia, headaches, decreased sensation and weakness of lower extremities	NS	NS
(51)	M	43	unilateral	No adrenelectomy	42	NS	Hypertension, tachycardia, headaches, pathologic hip and femur fractures, limited mobility, red face, lower extermity edema	Chemotherapy (cyclophosphamide, vincristine, dacarbazine, zoledronic acid)	Hematoxylin, eosin
(52)	F	33	NA	No adrenalectomy	33	T3	Hypertension, tachycardia, decreased muscle strength of lower limbs, hypoesthesia below T7, increased tendon reflexes	Biopsy, Abandoned resection	NS
(53)	F	27	unilateral	27	40	L2 body	Hypertension, cauda equina syndrome	Emergency decompression, resection	NS
(54)	F	NS	unilateral	no adrenalectomy	34	L4, L5	Tachycardia, sweating, suspected preclampsia, spine and shoulder pain	Fusion, chemotherapy	NS
(55)	M	32	NS	NS	38	NS	Pain	Percutaneous vertebroplasty	NS
(55)	M	42	NS	NS	48	NS	Pain, neurological deficits	Decompression, partial resection	NS
(55)	F	47	NS	NS	49	C2	Pain	Resection	NS
(55)	M	50	NS	NS	51	T2	Hypertension, pain, neurological deficits	Decompression, partial resection	NS
(55)	F	55	NS	NS	63	L4	Hypertension, pain, neurological deficits	Decompression, partial resection	NS
(55)	M	30	NS	NS	37	T12	Pain	Partial resection	NS
(55)	M	57	NS	NS	62	T5	Hypertension, pain, neurological deficits	Decompression, partial resection	NS
(55)	M	46	NS	NS	70	C1	Hypertension, pain	Partial resection	NS
(55)	F	42	NS	NS	44	T4	Neurological deficits	Decompression, partial resection	NS
(55)	F	42	NS	NS	46	L1	Pain, neurological deficits	Decompression, partial resection	NS
(56)	M	38	Unilateral	38	41	L3 body	Lower back pain, numbness and decreased muscle strength and sensation in lower extremities	Total en-bloc spondylectomy, implantation of a patient-individual 3D-printed artificial vertebral body, stabilization	Synaptophysin and chromogranin A, negative cytokeratin, epithelial membrane antigen, other markers were negative
(57)	M	NS	NS	11	13	T3	Lower limb mobility disorder	Resection, titanium filled with bone allograft	NS
(57)	M	NS	NS	NS	58	S1, S2	Sacrococcygeal pain	Resection, pedicle screws	NSE
(57)	M	NS	NS	22	40	T3	Lower limb mobility disorder	Resection, titanium filled with bone cement, radiotherapy	NS
(57)	M	NS	NS	23	42	L1, L2	Lower limb mobility disorder	Resection, pedicle screws, radiotherapy	Inhibin a, SF-1 and Syn;
(58)	F	36	Bilateral	36	44	Axial skeleton	Hypertension, tachycardia, cafe au lait spots, palpitations, generalized body pain, dizziness, presyncope	Symptomatic treatment	NS
(6)	M	NS	Unilateral	64	65	C6	Hypertension, sweating, neck pain, left arm and leg weakness with paresthesia, and urinary incontinence	Preoperative embolization, laminectomy, resection, posterior spinal fusion	NS
(59)	M	46	Unilateral	46	46	T1-T2 body, T12 arch	Right-sided abdominal pain	NS	CgA
Presented case	M	43	Unilateral	No adrenalectomy	44	L1	Tachycardia, hypothyroidism, lower back pain	Laminectomy, corpectomy, stabilization, fixiation	Synaptophysin, IMSM1, Vimentin, negative AE1/3, S-100, SOX10, TTF1, Inhibin, CD10, Ki67 ~ 15%

## Discussion

Pheochromocytoma metastases to the brain and spine are exceptionally rare, representing uncommon destinations for the malignant spread of NETs. This systematic review encompassed 35 studies from 1977 to 2024 (60 spinal cases) and 18 studies from 1948 to 2022 (18 cranial cases), along with one case report, consolidating sparse data to highlight distinct clinical, diagnostic, and therapeutic challenges of CNS involvement in malignant (mPCC) (1). Unlike more frequent metastatic sites, such as the liver or non-spinal skeleton, brain and spinal metastases pose unique complexities due to their potential to impair neurological function, disrupt spinal stability, or both (1). These challenges are exemplified by the case of a 41-year-old male with right frontoparietal brain metastasis causing progressive left-sided weakness. This case underscores the need for heightened diagnostic vigilance and tailored management strategies to address this rare metastatic site (1).

### Clinical presentation and symptom variability

The clinical presentation of mPCC metastases varies significantly by site, reflecting the local anatomical and physiological impacts. Cranial metastases, as seen in our case and reported in 18 patients, predominantly manifest with neurological symptoms such as headaches (44.4%, n=8) and focal deficits (e.g., described case’s weakness), with hypertension present in only 27.8% (n=5) and no reports of tachycardia or tremors. This neurological predominance, often associated with solitary lesions (55.6%, n=10), contrasts with the systemic catecholamine-driven symptoms typical of classical PCC. Spinal metastases, analyzed in 60 patients, more frequently present with hypertension (51.7%, n=31), headaches (13.3%, n=8), and tachycardia (10%, n=6), consistent with catecholamine excess (10). This dichotomy—neurological deficits in cranial cases versus structural and pain-related issues in spinal cases—emphasizes the need for clinicians to consider mPCC in PCC patients presenting with neurological symptoms or intractable back pain, even in the absence of classical features (10).

### Diagnostic challenges and imaging evolution

Diagnosing CNS mPCC metastases is challenging because of their rarity, nonspecific symptoms, and evolution of imaging modalities (5). Historically, spinal cases have often utilized non-contrast CT (41.7%, n=25) for its accessibility, despite its limited soft tissue resolution, compared to contrast-enhanced MRI, which accurately delineates epidural spinal cord compression and instability, guiding surgical planning (5). MRI (16.7%, n=10) and PET (21.7%, n=13) were underutilized in spinal cases but offered superior detailed and metabolic insights, as recommended by the current guidelines (60). For cranial metastases, MRI was critical in our case, although the review’s imaging data were incomplete, variably mentioning MRI and CT findings. The prolonged latency between initial PCC diagnosis and metastasis—11.6 years for cranial (mean age 46.6 years, SD 14.1) and 4 years for spinal (mean age 44.5 years, SD 16)—is reflected in the 17-year interval post-adrenalectomy (5). These extended intervals underscore the importance of long-term surveillance, prioritizing MRI for suspected CNS metastases to assess neurological and structural involvement, supplemented by CT for spinal bony anatomy, and PET for systemic staging (60). The historical reliance on CT and underutilization of advanced imaging highlights the diagnostic gap that modern protocols must address (9).

### Surgical management and outcomes

Surgical management remains the cornerstone of both cranial and spinal mPCC and is tailored to address site-specific deficits (6). The neuronavigation-assisted frontoparietal tumor resection with cranioplasty preserved neurological function, aligning with the review’s high surgical prevalence for cranial metastases (72.2%, n=13), often supplemented by radiotherapy (38.9%, n=7) or chemotherapy (11.1%, n=2) (6). The focal nature of spinal lesions (mean 1.7, SD 1.5) suggests amenability to targeted intervention, but our case’s prior recurrences emphasize the need for meticulous preoperative optimization to mitigate catecholamine surges, which is a critical consideration in CNS mPCC surgeries (6). Systemic therapies, such as 177Lu-DOTATATE in the described Case, and radiotherapy show promise for unresectable or recurrent disease, warranting further exploration (6).

### Histopathological insights and genetic context

Histopathologically, CNS metastatic pheochromocytoma (mPCC) consistently exhibits neuroendocrine marker expression. Described Case’s cranial lesion (Chromogranin A++, Synaptophysin++, Vimentin++) aligns with the systematic review’s findings, which reported Chromogranin A in 10 cranial cases and Synaptophysin in 7, confirming diagnostic consistency (2). However, the lack of comparative proliferation data, such as Ki67 levels, limits its prognostic utility. Genetically, hereditary syndromes are rare in cranial cases, with only 16.7% (n=3) showing predisposition and none linked to von Hippel-Lindau syndrome (2). Described in this study Case exhibited no genetic predisposition, consistent with most cases. The absence of SDHB mutation testing, a known malignancy risk factor, reflects a common gap in the historical data, restricting risk stratification (2, 3). Routine genetic profiling, particularly for SDHB, could identify patients at a higher risk of CNS metastases, thereby enhancing surveillance and management (3).

### Limitations and future directions

The rarity of cranial metastatic pheochromocytoma (mPCC) poses significant limitations, with a small sample size (18 cranial cases) and extended study periods (1944–2024), leading to incomplete reporting, including 33% of missing follow-up data (61). The historical preference for non-contrast CT over MRI or PET reduces diagnostic precision, whereas inconsistent genetic testing (e.g., SDHB) limits comprehensive risk assessment (9). These gaps underscore the need for prospective studies to standardize diagnostic and therapeutic protocols (61). Interdisciplinary collaboration—integrating neurosurgery, endocrinology, oncology, radiotherapy, and potentially plastic surgery for soft tissue reconstruction after spinal procedures—could optimize functional outcomes and quality of life (61). International registries and collaborative research are essential to address these uncommon metastatic sites and improve the detection, management, and survival in this rare malignancy.

## Conclusions

This systematic review and case report highlights the rarity of pheochromocytoma metastases to the brain and spine, emphasizing distinct clinical presentations (neurological for cranial and pain-driven for spinal), the critical role of MRI and PET in diagnosis, and the necessity of tailored surgical interventions. Despite diagnostic and data limitations, the findings advocate for long-term surveillance, advanced imaging, and multidisciplinary management to optimize outcomes in this challenging malignancy. Prospective studies and international registries are needed to standardize care and improve prognoses.

## Data Availability

The original contributions presented in the study are included in the article/**Supplementary Material**. Further inquiries can be directed to the corresponding author.
