# Correction: Pheochromocytoma metastasis to the central nervous system: a case report and systematic review

**DOI:** 10.3389/fendo.2025.1694463

**Published:** 2025-09-10

**Authors:** Justyna Małgorzata Fercho, Oskar G. Chasles, Jakub Chamier-Gliszczyński, Dominik Ryszard Płaza, Bogdan Jabłoński, Klaudia Kokot, Maciej Mielczarek, Małgorzata Małłek-Grabowska, Jacek Szypenbejl, Adrian Szkudlarek, Tomasz Szmuda, Mariusz Siemiński, Jacek Furtak

**Affiliations:** ^1^ Neurosurgery Department, 10th Military Research Hospital and PolyClinic SPZOZ, Bydgoszcz, Poland; ^2^ Department of Emergency Medicine, Medical University of Gdańsk, Gdańsk, Poland; ^3^ Lung Transplant Unit, Cardiac Surgery Department, Medical University of Gdańsk, Gdańsk, Poland; ^4^ Scientific Circle of Neurotraumatology, Department of Emergency Medicine, Medical University of Gdańsk, Gdańsk, Poland; ^5^ Neurosurgery Department, Stanisław Staszic Specialist Hospital, Piła, Poland; ^6^ Department of Anesthesiology and Intensive Therapy, 10th Military Research Hospital and Polyclinic SPZOZ, Bydgoszcz, Poland; ^7^ Faculty of Medicine, Bydgoszcz University of Science and Technology, Bydgoszcz, Poland; ^8^ Faculty of Medicine, Medical University of Gdańsk, Gdańsk, Poland

**Keywords:** pheochromocytoma, malignant pheochromocytoma, spinal metastases, cerebral metastases, central nervous system

Affiliation “Faculty of Medicine, Medical University of Gdańsk, Gdańsk, Poland” was omitted for author “Tomasz Szmuda”. This affiliation has now been added for author “Tomasz Szmuda”.

Author “Tomasz Szmuda” was erroneously assigned to the affiliations “Lung Transplant Unit, Cardiac Surgery Department, Medical University of Gdansk, Gdansk, Poland” and “Neurosurgery Department, Medical University of Gdansk, University Clinical Centre in Gdansk, Gdansk, Poland”. These affiliations have now been removed for author “Tomasz Szmuda”.

The original version of this article has been updated.

