# Supplementary figures and images for: Pheochromocytoma metastasis to the central nervous system: a case report and systematic review
Source: Front Endocrinol (Lausanne). 2025 Aug 25;16:1633411. doi: 10.3389/fendo.2025.1633411 (PMC12414785; doi:10.3389/fendo.2025.1633411)

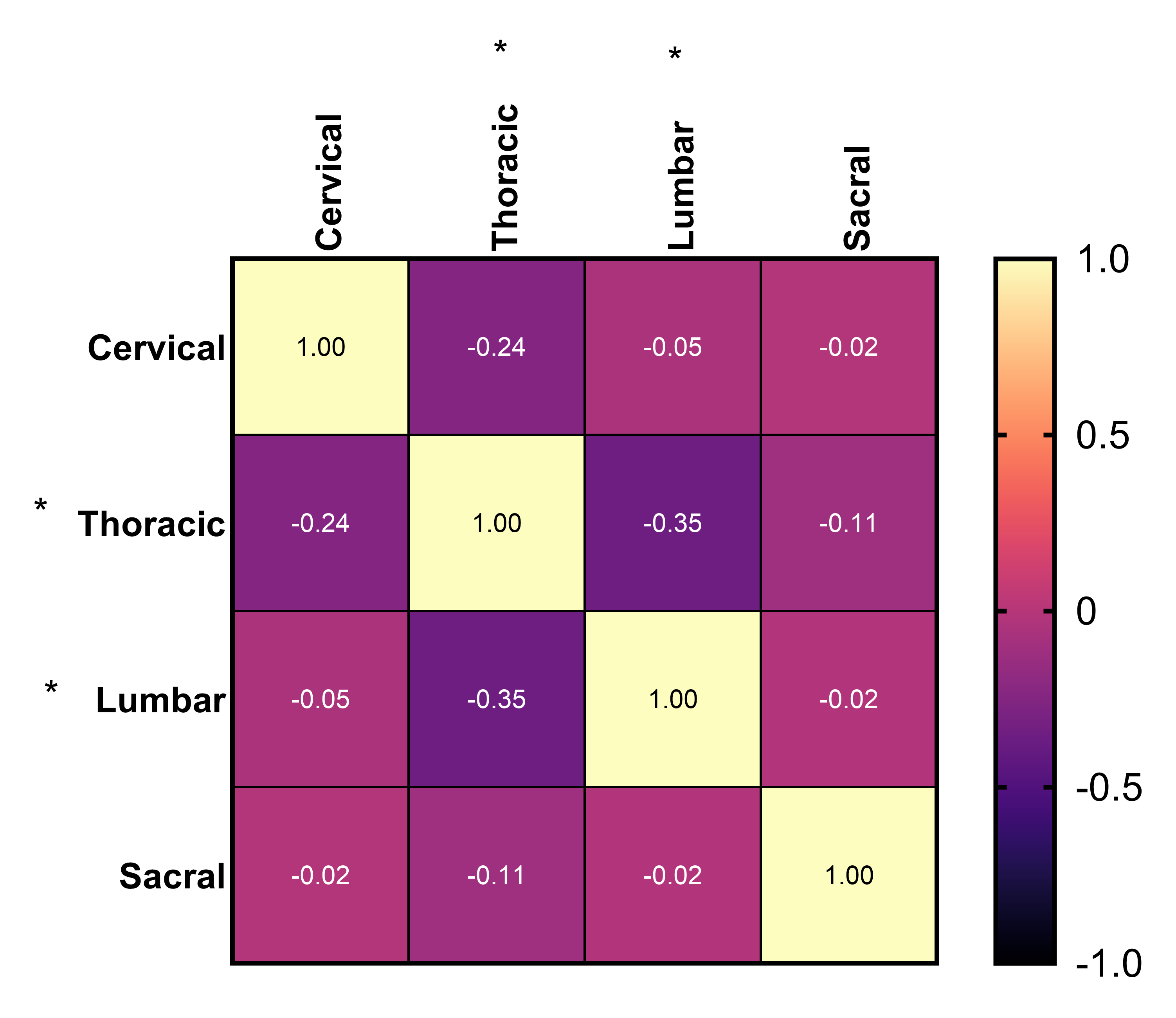

Supplement: Supplementary file 1 [file Image1.png]
